# Transient Erythroblastopenia of Childhood: An Atypical Presentation

**DOI:** 10.7759/cureus.100958

**Published:** 2026-01-06

**Authors:** Srushti Patel, Amit Shrestha, Marsha Medows

**Affiliations:** 1 Pediatrics, Woodhull Medical Center, New York, USA; 2 Pediatrics, New York University School of Medicine, New York, USA

**Keywords:** anemia, blood transfusion, normocytic anemia, pediatric anemia, reticulocytopenia, severe anemia, transient erythroblastopenia of childhood

## Abstract

Transient erythroblastopenia of childhood (TEC) is a self-limited, immune-mediated pure red cell aplasia typically affecting children under four years, characterized by normocytic anemia and reticulocytopenia. We report an atypical presentation in an older child with an unusual jaundice-like presentation due to lighting conditions, which underscores the importance of early reticulocyte count in evaluating pediatric anemia. Supportive transfusion and expectant management led to complete spontaneous recovery. Prompt recognition prevents unnecessary invasive testing and ensures optimal outcomes.

## Introduction

Transient erythroblastopenia of childhood (TEC) is a rare, self-limited pure red cell aplasia affecting ~1-20 per 100,000 children under four years annually (peak six to 36 months) [1,2]. It shows slight male predominance and possible spring/summer seasonality [2]. Clinically, we suspect this in a previously healthy toddler with new-onset normocytic anemia (hemoglobin (Hb) 4-8 g/dL) and reticulocytopenia (<1%). Confirmatory diagnosis requires CBC, reticulocyte count, and blood smear, while excluding parvovirus B19 or Diamond-Blackfan anemia (DBA) [3]. When bone marrow aspiration is done, it shows absent erythroid precursors with normal myeloid/megakaryocytic lines. But such invasive intervention is rarely needed [4]. The management is largely supportive, with serial monitoring for Hb. Transfusion is done only for symptomatic Hb <6-7 g/dL. Prognosis is excellent, with spontaneous recovery in four to eight weeks marked by reticulocytosis. Our case had atypical presentation of age and severe pallor appearing as pseudo-jaundice.

## Case presentation

A previously healthy six-year-old male with no significant past medical history presented with a change in skin color and progressive weakness for one week. His parents noticed that he was “looking yellow,” which raised concern for jaundice. They also reported decreased appetite during the same period but denied fever, dark urine, abdominal pain, vomiting, or recent infections. He was born in Honduras after an uncomplicated pregnancy and delivery and has lived in the United States for the past four years. His immunizations were up to date. There was no family history of hematologic or liver disorders, and he was not on any medications. His vitals were within normal limits. On examination, the child appeared pale with a faint yellowish hue, most noticeable under indoor lighting, giving the impression of jaundice. His sclerae were anicteric, and there was no hepatosplenomegaly, lymphadenopathy, or petechiae. Cardiac examination revealed a grade 2/6 soft systolic ejection murmur best heard at the left upper sternal border, without radiation or associated clicks or gallops. The remainder of the physical exam was unremarkable except for slight tachycardia. Given the initial concern for jaundice versus anemia, basic labs including a CBC and comprehensive metabolic panel (CMP) were obtained. The CBC revealed severe normocytic, normochromic anemia (Hb 4.7 g/dL, hematocrit (Hct) 13.2 %), with marked reticulocytopenia (0.2%) and normal white blood cell and platelet counts. Blood smear showed normocytic normochromic anemia with no abnormal blast cells. The CMP, bilirubin, and liver enzymes were within normal limits. Differential diagnosis included TEC and infectious diseases such as parvovirus and Epstein-Barr virus (EBV) given normocytic anemia and reticulocytopenia, but the serologies and PCR were negative. DBA was not initially suspected given the patient’s age, lack of congenital anomalies, and previously normal health. Aplastic anemia was less likely given the lack of pancytopenia.

Transferrin, iron, total iron-binding capacity, and ferritin were within standard values. Vitamin B12 and folic acid data were unremarkable and thus less concerning for a nutritional deficiency.

Slight elevation of lactate dehydrogenase (LDH), normal haptoglobin, normal uric acid, negative Coombs, normal total bilirubin, no hepatosplenomegaly, and reticulocytopenia made it less concerning for hemolysis. WBC counts were normal, ruling out any leukemia. Given the patient’s immigrant background, hemoglobin electrophoresis was obtained to evaluate for possible hemoglobinopathy and demonstrated a normal hemoglobin pattern. Given these values and the differential diagnoses for his anemic symptoms, the patient was started on slow packed RBC (pRBC) transfusions at 5 mL/kg over two hours to minimize the risk of heart failure. He received a total of 15 mL/kg of pRBCs, divided into three aliquots over two days. Post-transfusion laboratory values showed improvement. Hemoglobin and hematocrit increased on follow-up with hematology at 15 days and normalised by three months, with corresponding reticulocyte recovery, as shown in Table 1.

**Table 1:** Laboratory values with reference range shown at presentation, immediately post-transfusion, at the first follow-up with hematology-oncology (15 days after discharge), and at the second follow-up (three months after discharge). RDW: red blood cell distribution width, MCH: mean corpuscular hemoglobin, MCV: mean corpuscular volume, MCHC: mean corpuscular hemoglobin concentration

Component	Reference range	Day of Presentation	Post-Transfusion	First Follow-up	Second Follow-up
Hemoglobin	11.5 - 15.5 g/dL	4.7	8.0	9.1	13.3
Hematocrit	35.0 - 40.0 %	13.2	22.7%	28.5%	39.2%
Reticulocyte	% 0.5 - 2.0 %	0.2 %	0.4%	10.7%	1.8%
Reticulocyte Abs	0.050 - 0.100 x10(6)/mcL	0.003	0.012	0.355	0.091
RDW	11.5 - 13.4 %	11.5%	12.4%	18.6%	11.9%
MCV	77.0 - 95.0 fl	78.1	74.9	85.8	80.0
MCH	25.0 - 33.0 pg	26.4	26.4	27.4	27.1
MCHC	31.0 - 37.0 g/dL	33.8	35.2	31.9	33.9
WBC	4.50 - 13.50 x10(3)/mcL	5.04	6.14	4.49	6.04
Platelets	150 - 440 x10(3)/mcL	367	326	311	207

## Discussion

The initial concern for jaundice in this patient reflected the visual similarity between pallor and icterus, especially under certain lighting conditions or in children with fair or olive skin tones. However, the absence of scleral icterus and the normal bilirubin levels directed attention toward severe anemia as the cause of the altered skin color.

TEC is a rare, self-limited pure red cell aplasia characterized by normocytic normochromic anemia and profound reticulocytopenia [1]. The condition is generally benign, with spontaneous hematologic recovery within one to two months. It is considered a diagnosis of exclusion after ruling out infectious, congenital, nutritional, and malignant causes. This case of a six-year-old previously healthy boy presenting with progressive pallor misperceived as jaundice, severe anemia (hemoglobin 4.7 g/dL), and reticulocytopenia (0.2%) without fever, organomegaly, or hemolysis exemplifies classic TEC features while highlighting an atypical older age at presentation and the diagnostic challenge posed by the initial jaundice-like appearance under indoor lighting.

The pathophysiology of TEC remains incompletely understood but is widely regarded as immune-mediated, involving transient suppression of erythroid precursors in the bone marrow, often triggered by a preceding viral illness in approximately 50% of cases, though this case has no preceding illness [2,3]. Negative serologies and PCR for parvovirus B19 and EBV, common mimics, appropriately excluded active infection. The absence of hemolysis - evidenced by normal total bilirubin, haptoglobin, LDH (mildly elevated), and negative Coombs test - along with normal iron studies, vitamin B12, folate, and hemoglobin electrophoresis, further supported a non-destructive, non-nutritional, and non-hemoglobinopathic etiology in our patient. The patient’s Honduran origin prompted electrophoresis to rule out thalassemia or sickle cell variants [4]. The slight tachycardia and grade 2/6 systolic flow murmur were consistent with high-output compensation for severe anemia and resolved post-transfusion, highlighting the cardiovascular burden of untreated profound anemia.

Differential diagnosis in a child with isolated erythroid failure must systematically exclude DBA, aplastic anemia, leukemia, and transient viral suppression. DBA was unlikely given the patient’s age beyond infancy, absence of congenital anomalies, and spontaneous recovery without steroid therapy - hallmarks to differentiate the two [5]. Aplastic anemia and leukemia were excluded by normal leukocyte and platelet counts, absence of blasts, and lack of pancytopenia. The diagnostic hallmark in this case was the brisk reticulocyte response (0.4% to 10.7%) within 15 days, confirming marrow recovery and ruling out the need for bone marrow biopsy [5-7].

Management was appropriately supportive, with slow pRBC transfusions (5 mL/kg aliquots over two hours, total 15 mL/kg over two days) to correct symptomatic anemia and prevent high-output cardiac failure. No corticosteroids or immunosuppressive agents were indicated, unlike in DBA. Serial monitoring demonstrated progressive normalization: hemoglobin 9.1 g/dL and reticulocytes 10.7% at 15 days, and full recovery (hemoglobin 13.3 g/dL, hematocrit 39.2%, reticulocytes 1.8%) by three months - a timeline consistent with the natural history of TEC, where median recovery occurs within four to eight weeks and recurrence is rare [8,9].

## Conclusions

This case is notable for its presentation in a six-year-old, beyond the typical peak age of one to three years, and for the absence of a clear viral prodrome or familial clustering. The misperception of pallor as jaundice under artificial lighting highlights a common diagnostic pitfall and underscores the importance of thorough physical examination and early complete blood count in children with color change. The patient’s immigrant background further emphasizes the need for culturally informed evaluation, including hemoglobinopathy screening when relevant. Ultimately, this report reinforces TEC as a reversible cause of severe anemia requiring only supportive care and close follow-up, with excellent prognosis when promptly recognized and managed conservatively.
